# Expression of Ice Nucleation Protein in *Bacillus amyloliquefaciens* and Its Application in Food Freezing Process

**DOI:** 10.3390/foods12213896

**Published:** 2023-10-24

**Authors:** Rong Song, Cong Jiang, Jing Zhu, Jia Liu, Li Zhang, Jingnan Zuo, Wei Zheng, Shilin Liu, Qingrong Huang, Xuetuan Wei, Yijie Chen

**Affiliations:** 1 Key Laboratory of Environment Correlative Dietology, Ministry of Education, Wuhan 430070, China; songrong@mail.hzau.edu.cn (R.S.); jiangcong409@163.com (C.J.); spzjn2017@163.com (J.Z.); zhengwei98@webmail.hzau.edu.cn (W.Z.); slliu2013@mail.hzau.edu.cn (S.L.); weixuetuan@mail.hzau.edu.cn (X.W.); 2 College of Food Science and Technology, Huazhong Agricultural University, Wuhan 430070, China; 3 Hubei Institute of Measurement and Testing Technology, Wuhan 430070, China; zhujing_0711@163.com; 4 College of Life Science, Yangtze University, Jingzhou 434023, China; 18402814620@163.com; 5 Department of Food Science, Rutgers University, New Brunswick, NJ 08901, USA; zhanglinjau@gmail.com (L.Z.); qhuang@sebs.rutgers.edu (Q.H.)

**Keywords:** *Bacillus amyloliquefaciens*, frozen food, ice nucleation activity, ice nucleation proteins (INPs), recombinant protein expressing

## Abstract

To produce food-grade ice nucleators, a 3.77 kb ice nucleation gene (*iceE*) isolated from *Pantoea agglomerans* (*Erwinia herbicola*) was introduced into the Gram-positive microorganism *Bacillus amyloliquefaciens* for the first time. The differential scanning calorimetry (DSC) results indicated that recombined strain B9-INP was an effective ice nucleator for controlling the supercooling point of distilled water at low concentrations. In the presence of B9-INP cells, model food systems, including sucrose solution and sodium chloride solution, different pH solutions froze at a relatively high subzero temperature, thus increasing the supercooling point by 5.8~16.7 °C. Moreover, B9-INP also facilitated model and real food systems to freeze at −6 °C. This recombinant strain not only improved the freezing temperature of food systems but also shortened the total freezing time, thus saving energy and reducing consumption. The results suggest that B9-INP has great application potential in the frozen food industry.

## 1. Introduction

In the field of cloud physics, the process of ice nucleation assumes pivotal significance, as it holds extensive implications not only in the realm of biology, medicine, and food processing but also in various other disciplines [1]. In the absence of ice nucleating particles, the formation of ice in the atmosphere is limited to temperatures as low as −38 degrees [2]. Ice nucleating particles can originate from diverse sources, encompassing desert dust, marine aerosols, as well as the surfaces of terrestrial and marine plants, fungi, and animals [3,4,5]. Research findings indicate that biological materials possess significant ice nucleation activity, with only a minority demonstrating the ability to expedite ice nucleation [6,7]. These include various organisms such as bacteria, fungi, plants, insects, and algae [8,9,10]. At relatively high temperatures below zero, ice nucleation proteins (INPs) have the remarkable ability to catalyze the conversion of water into ice. INPs are synthesized by a diverse range of Gram-negative microorganisms like *Xanthomonas*, *Pantoea agglomerans* (formerly *Erwinia herbicola*), and *Pseudomonas*. Previous studies have indicated that a beta-helix structure within the central repeat domain of INPs plays a critical role in regulating ice nucleation [6]. The current research is centered on elucidating the mechanism underlying the molecular-level processes responsible for the formation of bacterial ice nucleation.

Freezing is one of the best available methods for long-term food preservation. Water could be removed from the food matrix by freezing and forming ice crystals [11]. When frozen foods are slowly frozen, large ice crystals will form in the extracellular regions, thus damaging the food microstructure and affecting its quality [12]. Previous studies have shown that the addition of INP-expressing bacteria and extracellular ice nucleators (ECINs) during freezing into model foods and real foods can shorten the freezing time, increase the ice nucleation temperature [13,14,15,16], and improve the quality of frozen foods [17,18]. Using extracellular ice nucleators, fish gels can be protected against freezing degeneration by stabilizing actomyosin [19,20]. The utilization of INPs obtained from *Pantoea agglomerans* has been found to enhance the viability of yeast and improve the baking quality of frozen dough [21]. Adding *Pseudomonas syringae pv. panici* to tylose gel/water-based solid model food meant that the microstructure was well modified [16]. The application research of INPs in liquid food is more extensive. In addition, the use of INPs exhibits multiple advantages in freeze drying and freeze concentration, such as energy saving and consumption reduction. Studies have shown that INPs can alter the ice morphology to facilitate the drying process, thereby resulting in a faster primary drying rate and a shorter total drying time [22]. By adding INPs to the desalting cycle to obtain fresh water, about 50% of the energy costs related to the process of freezing concentration can be saved [23]. The utilization of INPs shows a promising prospect for reducing energy expenditure, ultimately making food processing activities more economically feasible. Previous studies have demonstrated that the utilization of INPs in frozen food offers two significant benefits. Firstly, it enables energy conservation by elevating the ice nucleation temperature and modifying the ice structure. Secondly, it safeguards the frozen food’s quality by stabilizing its microstructure.

It is widely acknowledged that bacteria capable of initiating ice nucleation are predominantly plant pathogens or enterobacteria [24], and thus, the application of bacterial nucleators to the food industry arouses serious concerns about safety and toxicity. In addition, the expense of cell culture and the complex process of protein extraction pose large barriers to the widespread application of bacterial nucleators in food production. Expressing the ice nucleation gene in non-pathogenic and consumer-friendly microbial hosts represents a viable solution. *Bacillus*, typically regarded as safe for consumption (GRAS), represents an optimal host for producing food-grade ice nucleators. *Bacillus amyloliquefaciens*, a Gram-positive aerobic bacterium frequently present in soil, possesses an extensive repertoire of genetic tools, promoters, and plasmid expression systems, rendering it a valuable asset in the domains of synthetic biology, metabolic engineering, and diverse heterologous protein expression. Due to the absence of exotoxins or endotoxins, *Bacillus amylolyticus* is generally regarded as a strain safe for consumption, thus finding extensive application in the manufacture of food enzymes. The primary focus of recent research has been on the optimization of various factors, including signal peptides, transport channel levels, chaperone protein levels, and promoters within expression and transport systems. These efforts aim to improve the production of alloproteins in *Bacillus amyloliquefaciens* [25].

In this study, we expressed *iceE* gene isolated from *Pantoea agglomerans* in the Gram-positive microorganism *Bacillus amyloliquefaciens* (BAX-9) for the first time. We investigated the ice nucleation activity of recombinant strain B9-INP in the freezing process. We evaluated the influence of the concentrations of cells, sucrose, and sodium chloride solution, as well as pH values on the ice nucleation activity of B9-INP in different liquid model systems and real foods. These findings provide a theoretical basis and practical references for the application of INPs in frozen food.

## 2. Materials and Methods

### 2.1. Strains and Culture Conditions

*E. coli* DH5α [*sup*E44 Δ*lac*U169 (f80 *lacZ*ΔM15) *hsdR*17 *recA*1 *gyrA*96 thi1 *relA*1] cells were selected as the recipient bacteria for DNA manipulations. DH5α was routinely grown in an LB broth (containing 10 g of bacto-tryptone, 5 g of bacto-yeast extract, and 5 g of NaCl per liter) with 20 µg/mL tetracycline. *Pantoea agglomerans* (acquired from the American Type Culture Collection (ATCC Cat. No. 27155)) was used as the parent strain for the *iceE* gene cloning. *Bacillus amyloliquefaciens* (BAX-9) was used for transformation experiments. BAX-9 harboring plasmid pHY300PLK (B9-INP) was grown in an LB broth. Plasmid pHY300PLK was the shuttle plasmid of *E. coli* and *Bacillus amyloliquefaciens.* The chromosomal DNA of *Bacillus subtilis* 168 (*B. s* 168) and *Bacillus licheniformis* (WX-02) was used as the promotor and terminator template, respectively.

### 2.2. Construction of Bacillus amyloliquefaciens Expression Plasmids and Transformation

The P43 promoter was amplified from *B. s* 168 chromosomal DNA using primers sP43-F-BamHI and sP43-INP-R. The Tamyl terminator was amplified from WX-02 chromosomal DNA using primers sT-INP-F and sT-R-XbaI. A 3777 bp ice nucleation gene (*iceE*) was cloned from the total genomic DNA of *Pantoea agglomerans* using primers sINP-P43-F and sINP-T-R. The linearization of pHY300PLK vector was performed by *Bam*HI and *Xba*I restriction enzyme double digestion. Then, the three PCR fragments were gel purified and ligated by the one-step cloning kit [26], and recombined plasmids were transformed into *E. coli* DH5α. Tetracycline-resistant positive transformants were further verified by colony-PCR and sequencing. Recombinant plasmid pHY300-INP was electrotransformed into BAX-9.

### 2.3. Bacillus Amyloliquefaciens BAX-9 Expression Analysis

B9-INP containing the recombination plasmids pHY300-INP was grown in an LB broth at 37 °C with shaking at 200 rpm until a density of 10^8^ CFU/mL. BAX-9 was used as a control. The culture broth was centrifuged at 6000 r/min for 15 min, and bacteria were collected and dissolved in distilled water to prepare bacterial suspension. The resultant bacterial suspension was freeze-dried for ice nucleation activity assays and subsequent experiments.

### 2.4. Determination of the Supercooling Point Using Differential Scanning Calorimetry

The determination of the supercooling point of bacterium solutions with different concentrations was conducted using a 200F3 differential scanning calorimeter (Netzsch Group, Selb, Germany) powered by liquid nitrogen and compressed nitrogen gas. The B9-INP was serially (10-fold) diluted in distilled water from 10 mg/mL to 10^−5^ mg/mL. Briefly, a 10 μL bacterium solution sample was sealed in a standard aluminum pan, with an empty pan as the control. The DSC unit’s temperature was decreased from 4 °C to −25 °C at a rate of 1 °C/min. The temperature point corresponding to the highest heat flow observed is referred to as the supercooling point. The distilled water containing 10 mg/mL BAX-9 was used as the control. The experiments were performed in triplicates [23].

### 2.5. Ice nucleation Activity Assays in Liquid Model Food System

Sucrose solution and sodium chloride solution at different concentrations (0.1 mol/L, 0.3 mol/L, 0.5 mol/L, 0.7 mol/L, and 1 mol/L) and acid-based solution (pH 2~11) were prepared. The freeze-dried powder of B9-INP was added into each of the above 3 solutions to reach a final concentration of 1 mg/mL (according to the results of Section 2.4). After mixing, 10 μL samples were absorbed and determined by DSC. The temperature of the DSC unit was reduced from 4 to −25 °C at the rate of 1 °C/min [23]. An empty aluminum pan was utilized as a point of reference. There were 3 replicates for each sample, and the average of the 3 replicates was taken.

### 2.6. Measurement of Freezing Curves in Liquid Model Food System

Different concentrations (m/m) of sucrose solution (10 mL) and sodium chloride solution (10 mL) containing B9-INP at different concentrations utilized in this study were subjected to radial freezing by placing them in plastic centrifuge tubes immersed in a stationary cooling bath comprising 50% ethylene glycol. The centrifuge tube was inserted into the float board so that the liquid level of the sample was at the same level as the bottom surface of the float board. The thermometer probe was inserted into the centrifuge tube with one end of the probe in the middle of the sample solution during the measurement. Three temperature gradients were set as −6 °C, −8 °C, and −10 °C, and temperature changes were recorded until the solution temperature remained stable [15].

### 2.7. Freezing Curves of Real Food System

According to the above results, a constant temperature was set. The freezing temperature curves of orange juice, apple juice, egg white, whole egg, whole milk, and skimmed milk (each 10 mL) containing different B9-INP concentrations were plotted [15].

### 2.8. Microscopic Observation of Ice Crystal

The 20 μL Distilled water, 20 μL BAX-9 (1 and 10^−2^ mg/mL), and 20 μL B9-INP (1 and 10^−2^ mg/mL) samples were, respectively, placed on microscope slides (at −18 °C). The ice crystal structure was evaluated utilizing a 10× lens (0.25 N.A.) microscope (MShot, Guangzhou, China) equipped with a temperature-controlled system (MK2000, INSTEK, Boulder, CO, USA). The ice crystal structure images at the ice surface layer were captured [23].

### 2.9. Statistical Analysis

PCR primers were designed using Primer Premier 5 (5.00) software. The experiments were conducted with three replicates. The data were expressed as the mean ± SD of three replicates. The diagrams were plotted using Origin 2021 (9.8.0.200) software. SPSS Statistics 26 (26.0.0.0) was used for data processing and statistical analysis.

## 3. Results and Discussion

### 3.1. The Amplification of IceE Gene and Identification of Recombinant Clones

**Figure 1:**
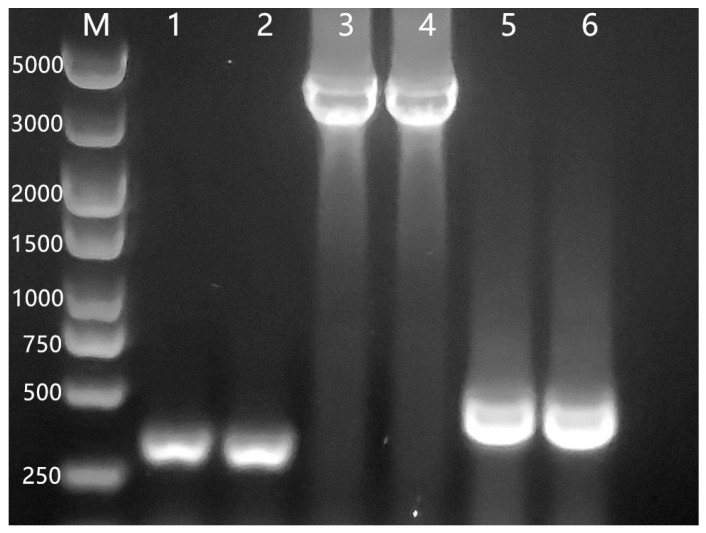
PCR amplification of promoter, *iceE* gene, and terminator. Lane M, 5 kb DNA ladder; lane 1–2, P43 promoter; lane 3–4, *iceE* gene; lane 5–6, Tamyl terminator.

**Figure 2:**
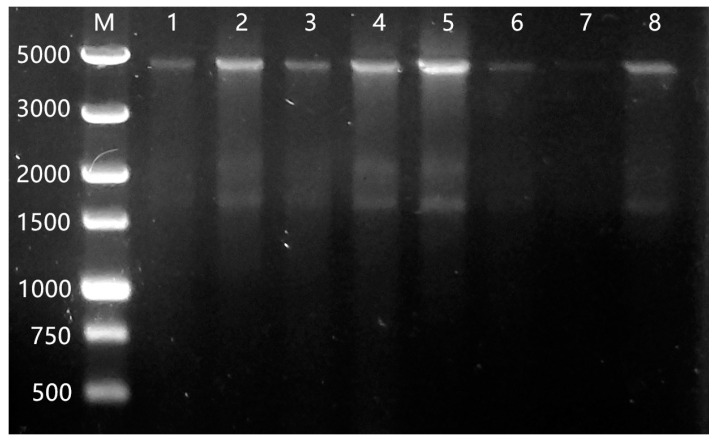
The result of colony-PCR. Lane M, 5 kb DNA ladder; lane 1–8, seven transformants with a predicted size.

The P43 promoter, *iceE* gene, and Tamyl terminator were cloned using primers via PCR. Recombination was verified using PCR colonies. As shown in Figure 1, the amplified bands corresponded to the expected PCR fragment size. Seven transformants with a predicted size of 4700 bp were selected (Figure 2). The sequencing analysis results of the recombinant plasmid pHY300-INP conformed with the anticipated results.

### 3.2. The Impact of B9-INP on Supercooling Point of Distilled Water

**Figure 3:**
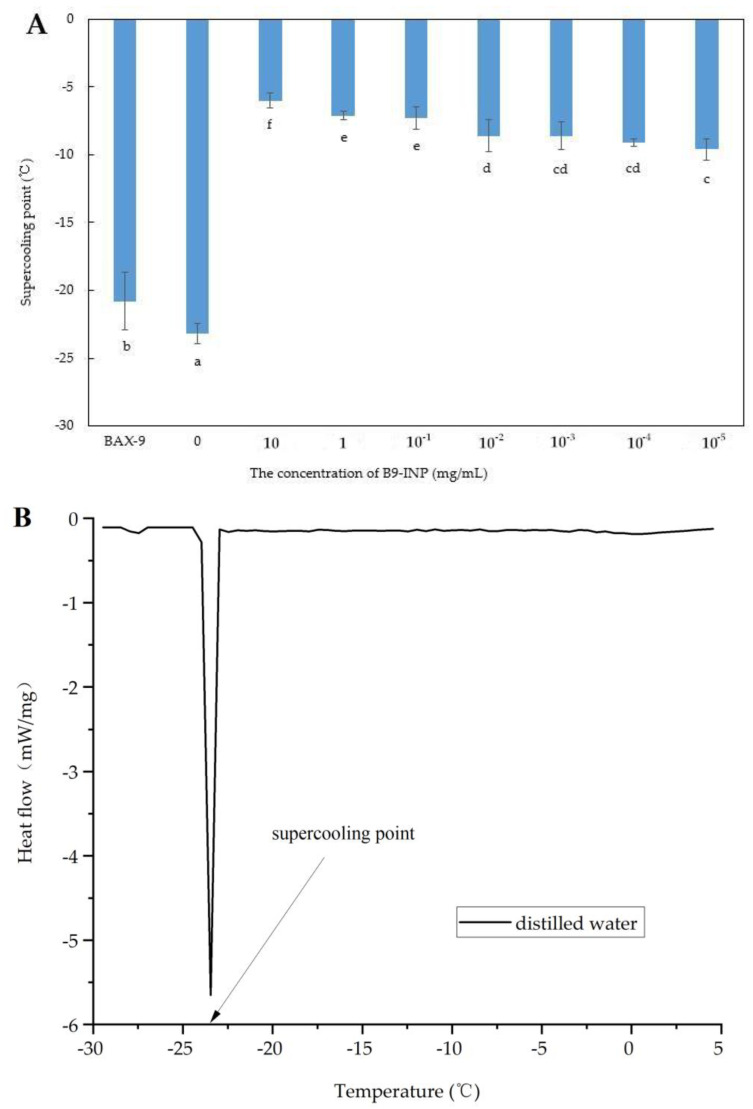
(**A**) Effect of increase in B9-INP concentrations on supercooling point of distilled water. The lower-case letters below the error bars signify significant differences (*p* < 0.05). (**B**) DSC thermogram of frozen distilled water at a cooling rate of 1 °C/min.

The effect of B9-INP on the supercooling point of distilled water was investigated using DSC (Figure 3A). The supercooling point refers to the temperature at which the phase transitions from water to ice of a supercooled solution, accompanied by the release of latent heat (Figure 3B). The results showed that the addition of B9-INP to reach a final concentration of 10 mg/mL resulted in an increase in the supercooling point of the samples to −6.0 °C, while that of the control and distilled water was −20.8 °C and −23.2 °C, respectively. The supercooling point of the samples added with B9-INP rose to −9.8 °C even at an additional concentration as low as 10^−5^ mg/mL. Previous research has indicated that the INP concentration is not linearly correlated with the supercooling point due to the impact of protein aggregation on ice nucleation activity [27]. From the initial investigations of *E. coli* to the recent software simulations, the formation of dimers and higher-order oligomers has been reported to be indispensable for INPs’ activities [6,28,29,30,31]. An aggregation process of this nature is not limited by the concentration range of INPs available. The 10 mg/mL B9-INP cells and 1 mg/mL INPs purified protein possess similar supercooling points [23]. However, the advantage of B9-INP lies in that it can function directly without additional protein extraction and purification, making the production of B9-INP easier. This study made the first attempt to express *iceE* gene in the Gram-positive bacterium B9-INP. Our results suggested that B9-INP exhibited significant efficacy as an ice nucleator in regulating the supercooling point of distilled water, even when present in low concentrations. In recombinant hosts, the threshold temperature for ice nucleation was usually lower than that in wild-type bacteria. The ice nucleation activity of B9-INP was only slightly lower than that of *E. coli*. One possible explanation is that Gram-positive bacteria have significantly different lipid compositions of the membranes from those of *E. coli* [32]. Furthermore, a challenge that may restrict the application of bacterial ice nuclei in food is the inherent characteristics of the microorganism that naturally produces ice nuclei. The vast majority of naturally existing bacteria that are capable of activating ice nucleation activity are Gram-negative. Although none of these ice nucleation activity organisms have demonstrated toxicity in mammals thus far, the presence of specific compounds associated solely with Gram-negative bacteria, such as lipopolysaccharides, could complicate the standard methods used to detect bacterial contamination in food. However, by selecting *Bacillus amyloliquefaciens*, this issue can be circumvented.

The increase in nucleation temperature contributes to saving a great deal of energy during the freezing processes. Given the correlation between the bacterium B9-INP concentration and its ice nucleation effect, 1 mg/mL B9-INP was selected in subsequent experiments.

### 3.3. Effect of B9-INP on Supercooling Point of Liquid Model Food System

**Figure 4:**
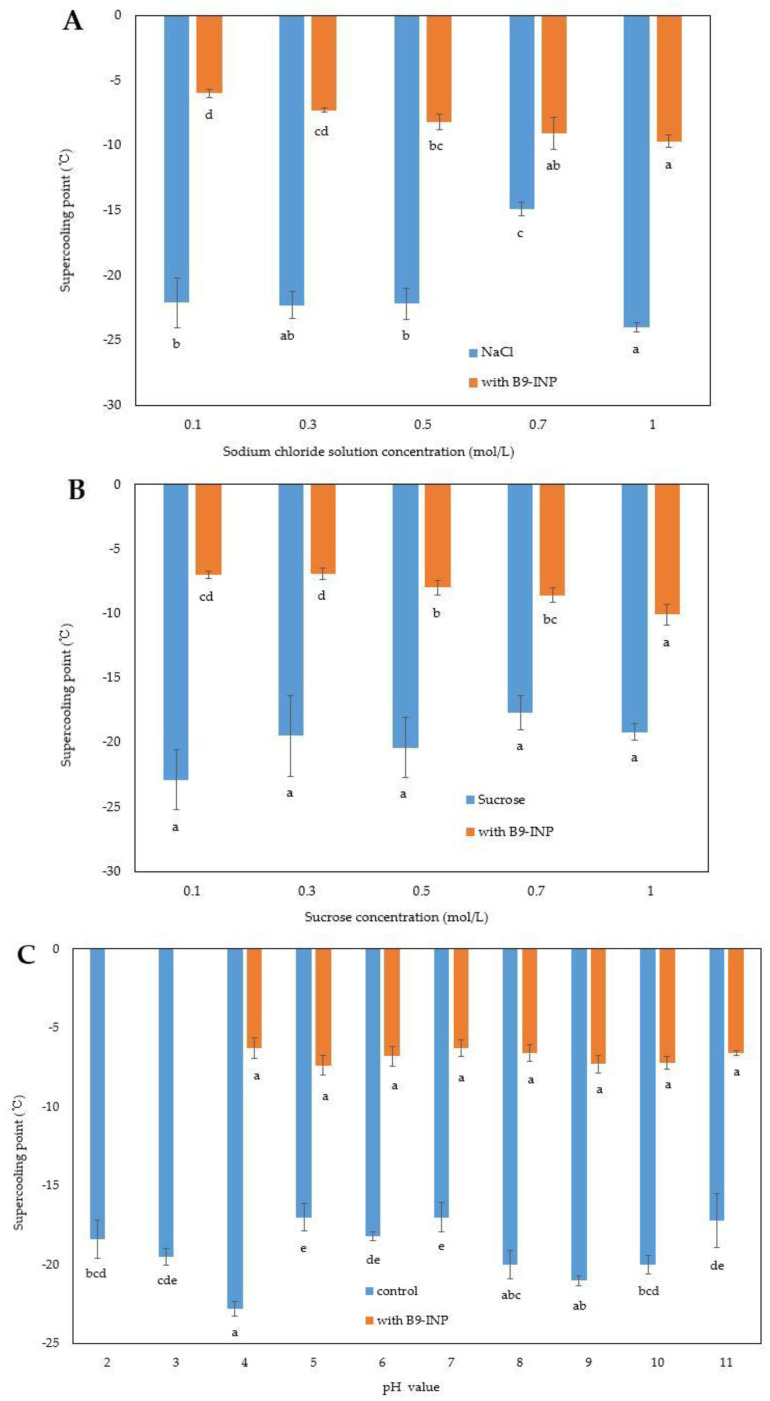
Effect of B9-INP on supercooling point of (**A**) sodium chloride solution, (**B**) sucrose solution, and (**C**) distilled water with pH values. The lower-case letters below the error bars signify significant differences (*p* < 0.05).

The impact of B9-INP on the supercooling point of sodium chloride solutions at various concentrations was explored through DSC (Figure 4A). The final concentration of B9-INP was 1 mg/mL. The results indicated that the supercooling point was decreased with the sodium chloride solution concentration increased. The addition of 1 mg/mL B9-INP resulted in an increase of 5.8~16.1 °C in the supercooling point in comparison to the control sample. Furthermore, even in a 1 mol/L sodium chloride solution, the supercooling point was still able to attain a high temperature. Similar to the changing trend of the supercooling point of sodium chloride solution, the supercooling point was gradually decreased with increasing concentrations of sucrose solution (Figure 4B).

Compared to that of the control, the supercooling point of distilled water added with 1 mg/mL B9-INP at different pH values was increased by 9.1~15.9 °C. Generally, adding any solute into a solution, whether it is organic or inorganic, will cause a decrease in its freezing point, preventing it from freezing. The nucleation of ice marks the time point when bulk water begins to freeze. Such a transformation might be attributed to the fact that INPs can efficiently promote ice formation by generating ice nucleators containing repetitive amino acid sequences. Previous studies of model foods have shown that with sucrose concentration increased, either by the addition of ice-nucleated bacteria or extracellular ice nucleators, the supercooling point decreased [13,14]. However, the effects of ice-nucleated bacteria or extracellular ice nucleators on sodium chloride solutions have not been studied yet. In this study, although the freezing point of solution added with B9-INP (−6~9.6 °C) was still lower than that of the solution added with other ice-nucleation bacteria (−2 °C for *P. syringae*; −2.5 °C for *P. ananatis*), B9-INP could still significantly increase the nucleation temperature of the model solution. Therefore, the addition of B9-INP can guarantee the freezing of some liquid foods at relatively high temperatures.

The effects of B9-INP on the supercooling point of solutions with different pH values were investigated (Figure 4C). The results showed that the addition of B9-INP did not cause any significant differences in the supercooling point among the B9-INP-containing solutions with different pH values. However, the supercooling point of the solutions containing B9-INP was 11.1~16.7 °C higher than that of the solutions containing no B9-INP (the control) at different pH values. It is noteworthy that bacterial INPs are less likely to form ice when they are exposed to acidic pH values [33,34]. A high supercooling point was observed not only in the solution containing living bacteria at acidic pH but also in the solution containing the non-viable bacterial product Snomax [34]. A white precipitate was observed at pH = 2 and 3, which suggested that bacteria tend to become inactive in a strongly acidic environment. Acidic pH might influence ice nucleation activity through the precipitation of more denatured protein complexes in living cells. Our engineering bacteria B9-INP exhibited good ice nucleation activity when the pH value ranged from 4 to 11.

### 3.4. Freezing Curves of Liquid Model Food System

**Figure 5:**
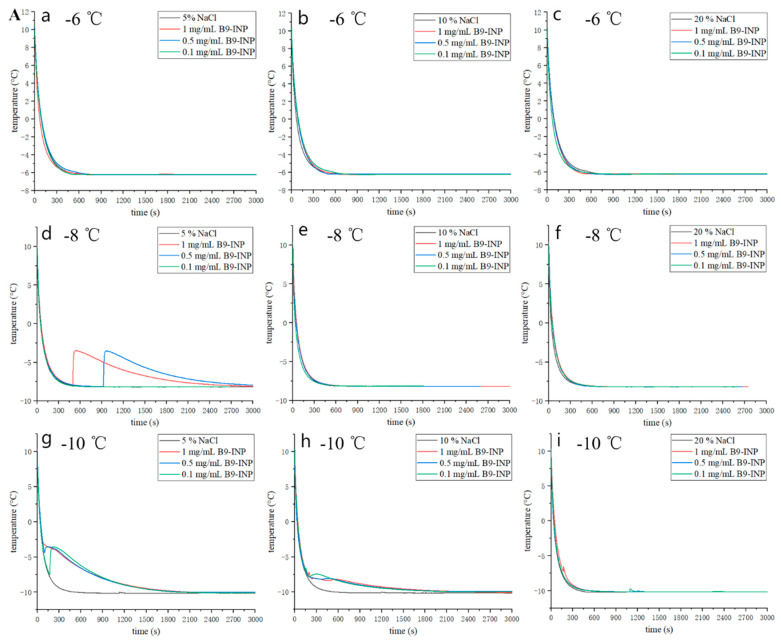

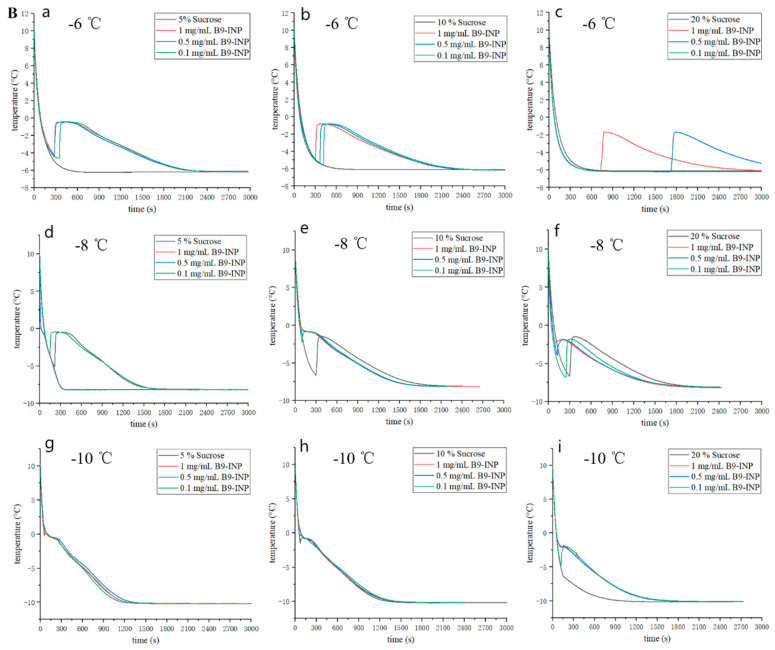
(**A**) Freezing curves of sodium chloride solution in the absence or presence of B9-INP. (**B**) Freezing curves of sucrose solution in the absence or presence of B9-INP. (**Aa**), −6 °C, 5% NaCl; (**Ab**), −6 °C, 10% NaCl; (**Ac**), −6 °C, 20% NaCl; (**Ad**), −8 °C, 5% NaCl; (**Ae**), −8 °C, 10% NaCl; (**Af**) −8 °C, 20% NaCl; (**Ag**) −10 °C, 5% NaCl; (**Ah**), −10 °C, 10% NaCl; (**Ai**), −10 °C, 20% NaCl; (**Ba**), −6 °C, 5% sucrose; (**Bb**), −6 °C, 10% sucrose; (**Bc**), −6 °C, 20% sucrose; (**Bd**), −8 °C, 5% sucrose; (**Be**), −8 °C, 10% sucrose; (**Bf**), −8 °C, 20% sucrose; (**Bg**), −10 °C, 5% sucrose; (**Bh**), −10 °C, 10% sucrose; (**Bi**), −10 °C, 20% sucrose.

The impact of B9-INP on the freezing process of sodium chloride solution and sucrose solution was investigated (Figure 5A). As shown in Figure 5A, none of the sodium chloride solutions were frozen. When the temperature reached −8 °C, ice nucleation activity was observed in 5% sodium chloride solution added with 0.5 mg/mL B9-INP (Figure 5Ad). As the temperature decreased to −10 °C, only 0.1 mg/mL B9-INP was required to freeze the same concentration (5%) of sodium chloride solution (Figure 5Ag). Moreover, with the concentration of B9-INP increased to 1 mg/mL, even a high concentration of sodium chloride solution (20%) could be frozen at −10 °C (Figure 5Ai).

In the sucrose solution group (Figure 5B), the freezing effect was superior. At a temperature of −6 °C, none of the concentrations of sucrose solutions was able to freeze without the addition of B9-INP. Sucrose solution with a high concentration (20%) was also unable to be frozen at −10 °C without the addition of B9-INP (Figure 5Bi). All concentrations of sucrose solutions were able to be frozen at −8 °C, but the sucrose solutions added with B9-INP froze faster or required a shorter total freezing time. Additionally, with the increasing cell concentration, the ice nucleation temperature was increased. It should be noted that high concentrations of sucrose solutions required a high concentration of B9-INP.

Many industrial processes rely on efficient freezing techniques, such as freeze storage, freeze drying, and freeze concentration. By utilizing subzero temperatures, it is possible to reduce freezing energy, increase productivity, and enhance the quality of various products [22,23]. This is particularly true with the food freeze-concentrate industry. Previous study has indicated that each degree decrease in freezing temperature results in energy consumption increases by approximately 6.5~8% [35,36]. Adding INPs can notably enhance the efficiency of block freeze concentration in seawater desalination models, thus significantly saving energy costs (reaching up to 50%) [23].

### 3.5. Freezing Curves of Real Food System

**Figure 6:**
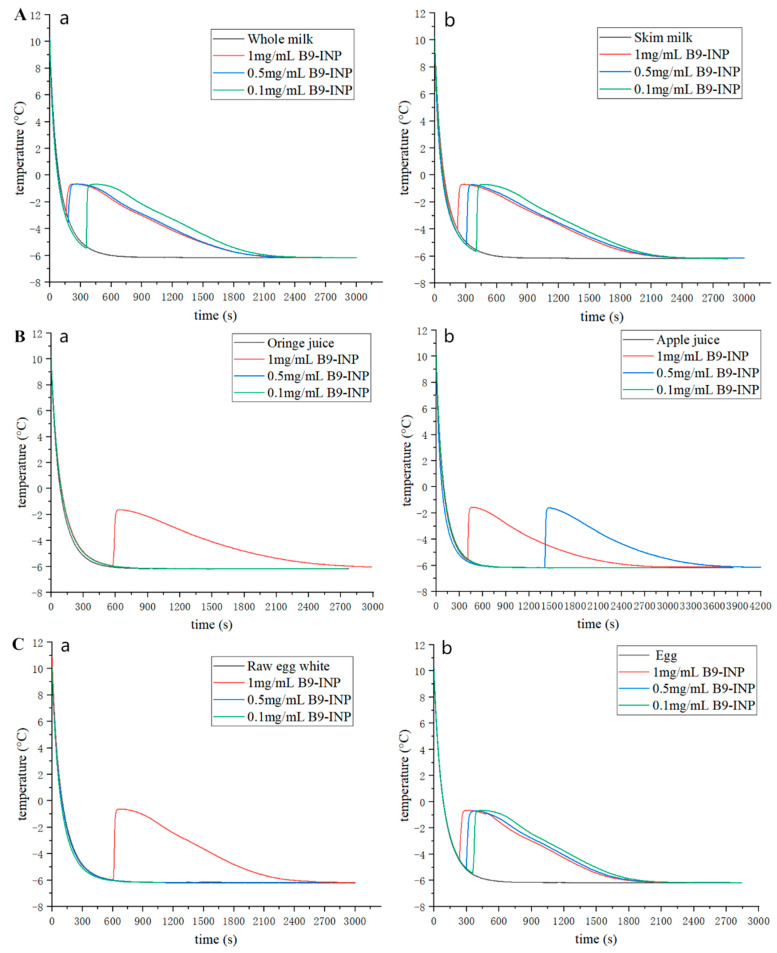
(**A**) Freezing curves of milk in the absence or presence of B9-INP. (**B**) Freezing curves of juice in the absence or presence of B9-INP. (**C**) Freezing curves of egg in the absence or presence of B9-INP. (**Aa**) whole milk; (**Ab**) skim milk; (**Ba**) orange juice; (**Bb**) apple juice; (**Ca**) raw egg white; (**Cb**) egg.

Some real foods were selected to test the ice nucleation activity of B9-INP. According to the results of the experiment in Section 3.4, the freezing temperature was set at −6 °C. In the milk group (Figure 6A), neither whole nor skimmed milk could freeze at −6 °C. After the addition of B9-INP, all the samples could be frozen, and the supercooling point of whole milk and skimmed milk was significantly increased. At 1 mg/mL B9-INP, the ice nucleation temperatures of whole and skimmed milk were −3.12 °C and −3.99 °C, respectively. The difference in supercooling points between the two groups of milk might be related to fat content. Research on ice cream has shown that the supercooling temperature was significantly increased with the increase in fat content (3~9%) in the process of making ice cream, which might be related to the increase in bound water content. As the fat content of the ice cream liquid increases (3~7%), a greater number of fat globules will form. Consequently, the non-flowing water trapped between these globules will progressively accumulate, leading to the gradual transformation of free water into non-flowing water [37]. In this study, the control groups of whole milk and skimmed milk exhibited basically the same freezing curve, which might be because there was no significant difference in their fat (lower than 3%).

In the juice group (Figure 6B), orange juice and apple juice did not freeze at −6 °C. At the concentration of B9-INP of 0.5 mg/mL for apple juice and 1 mg/mL for orange juice, two juices froze. This might be due to the difference in pH values as orange juice had a slightly lower pH than apple juice. Thus a higher concentration of B9-INP addition was required for orange juice.

In addition, the total freezing time of apple juice was 2594 s at the 1 mg/mL B9-INP and 3557 s at the 0.5 mg/mL B9-INP. The increase in the concentration of B9-INP saved nearly 27 percent of freezing time. The addition of 0.1 mg/mL of B9-INP could freeze whole egg liquid at −6 °C, but to freeze raw egg white required 1 mg/mL B9-INP, which might be due to the presence of the phospholipid in egg yolks. The ice nucleation activity genes have been successfully cloned and expressed in bacteria, zymomonas, and yeast so far [38]. Our recombinant bacterium B9-INP also showed excellent freezing-promoting ability in the food system.

### 3.6. Microscopic Observation of Ice Crystal

**Figure 7:**
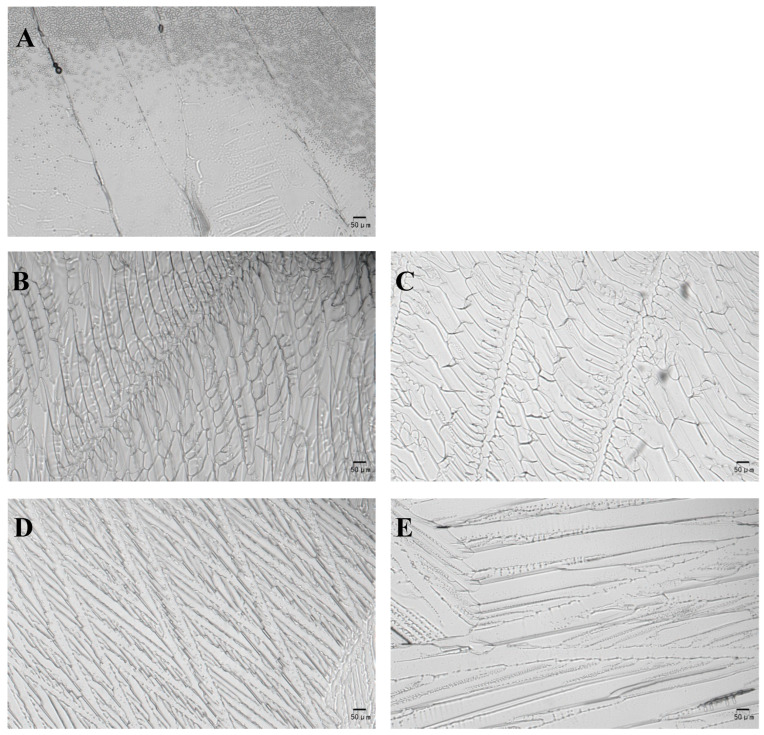
Ice formation patterns of distilled water, BAX-9 and B9-INP cell suspension. (**A**) Ice crystals formed by distilled water. (**B**) Ice crystals generated by 1 mg/mL BAX-9 suspension. (**C**) Ice crystals produced by 10^−2^ mg/mL BAX-9 suspension. (**D**) Ice crystals generated by 1 mg/mL B9-INP suspension (**E**) Ice crystals produced by 10^−2^ mg/mL B9-INP suspension.

The ice crystals of distilled water, BAX-9 suspension, and B9-INP suspension were characterized. The addition of B9-INP cells increased ice nucleation temperature, altered ice formation patterns, and produced a special ice crystal texture (Figure 7). Fine particles without any directionality were observed in distilled water (Figure 7A), where the ordered and directional textures were found in BAX-9 and B9-INP suspensions. However, the ice crystals formed by B9-INP were smaller than those formed by BAX-9 at the same concentration (Figure 7B,D). The ice crystals generated by B9-INP became larger with the decreased cell concentrations (Figure 7D,E), but the ice crystals produced by BAX-9 exhibited no correlation with the cell concentrations (Figure 7B,C).

In the food freeze concentration industry, a dry layer morphology greatly influences the heat transfer and ice sublimation rates during the primary drying process. Ice crystals formed during freezing determine the structure of porous layers [39]. In previous studies, researchers added ice nucleation proteins to the food system, and different freeze-dried cakes display lamellar ice structure along the growth direction and cross-section. INPs can enhance the freezing efficiency of liquid systems with various components. The possible reason might be that INPs have changed the ice morphology, thus leading to an increase in efficiency [22]. In addition, ice crystal formation is the main trigger factor for seafood damage during freezing [40]. The formation of ice crystals in the internal structure is an important factor affecting the quality of frozen cooked noodles [41]. The quality of frozen foods has been reported to be influenced by the size and distribution of ice crystals [12].

## 4. Conclusions

Bacterial ice nucleators exhibit great potential in the food processing industry. However, the application of bacterial ice nucleators is restricted in the food industry due to safety concerns over natural INP-expressing bacteria. To solve this problem, GRAS host cells can be adopted. In this study, we successfully expressed the *iceE* gene in *Bacillus amyloliquefaciens*. In the presence of B9-INP cells, model food systems, including sucrose and sodium chloride solutions, froze at a relatively high subzero temperature. B9-INP increased the supercooling point by 5.8~16.7 °C. In addition, B9-INP could also cause model and real food systems to freeze easily at −6 °C. This recombinant strain can not only increase the freezing temperature of the food system but also shorten the total freezing time, thus saving energy and reducing energy consumption. Our findings provide experimental data and theoretical support for the application of INPs in the frozen food industry.

## Data Availability

The data presented in this study are available on request from the corresponding author.
